# Trophic Neuralgias

**Published:** 1879-12

**Authors:** John J. Caldwell

**Affiliations:** Baltimore, Md.


### TROPHIC NEURALGIAS.

BY JOHN J. CALDWELL, BALTIMORE, MD.

By neuralgia of the pneumogastric and sympathetic we mean painful affections of the stomach and bowels, not dependent upon changes of structure, but simply hyperæsthesia of the pneumogastric and solar plexus, or gastrodynia and neuralgia colica.

CAUSES.—Anæmia, chlorosis, hysteria, onanism, and other neuroses, again dislocations, flexions, inflammations and ulcers of the uterus; affections of the ovaries, and may depend on diseases of the brain and spinal cord.

Like other neuroses, neuralgia of the pneumogastric and sympathetic are distinguished by their typical course, *i. e.*, after an interval of freedom, paroxysms of the severest pain occur, or suddenly after, a feeling of pressure there is a severe griping pain, extending to the back and throughout the bowels; a feeling of faintness, shrunken countenance, cold hands and feet, a small, intermittent pulse, pains so severe that the patient cries out, epigastrium puffed, or greatly retracted, the patient presses the pit of the stomach for relief.

In sympathetic neuralgia pains often occur in the thorax, under the sternum and along the œsophageal branches.

The attack lasts from a few minutes to half an hour, then gradually subsides, leaving the patient greatly exhausted, or else ceases suddenly with eructations of gas and watery fluid, with soft perspiration and reddish urine.

We lately lost a patient, a gentleman fifty years old, by sudden, severe attack of the above nature, termed, by some authorities angina pectoris. The feeling of faintness and impending death are fearful features of this trouble.

As an interesting neurosis in a child two years old we would mention as follows, a unilateral vaso-paresis of the right side, with mother's marks, *neavi-maternus*, in small blotches, scattered from head to foot, quite uniformly.

[TO BE CONTINUED.]

